# Unpacking analyses relying on area-based data: are the assumptions supportable?

**DOI:** 10.1186/1476-072X-3-30

**Published:** 2004-12-09

**Authors:** John Glover, Diana Rosman, Sarah Tennant

**Affiliations:** 1 Public Health Information Development Unit, The University of Adelaide, South Australia, Australia; 2 Health Information Centre, Department of Health, Royal Street, East Perth, Western Australia, Australia

## Abstract

**Background:**

In the absence in the major Australian administrative health record collections of a direct measure of the socioeconomic status of the individual about whom the event is recorded, analysis of the association between the health status, use of health services and socioeconomic status of the population relies an area-based measure of socioeconomic status.

This paper explores the reliability of the area of address (at the levels typically available in administrative data collections) as a proxy measure for socioeconomic disadvantage. The Western Australian Data Linkage System was used to show the extent to which hospital inpatient separation rates for residents of Perth vary by socioeconomic status of area of residence, when calculated at various levels of aggregation of area, from smallest (Census Collection District) to largest (postcode areas and Statistical Local Areas). Results are also provided of the reliability, over time, of the address as a measure of socioeconomic status.

**Results:**

There is a strong association between the socioeconomic status of the usual address of hospital inpatients at the smallest level in Perth, and weaker associations when the data are aggregated to larger areas. The analysis also shows that a higher proportion of people from the most disadvantaged areas are admitted to hospital than from the most well-off areas (13% more), and that these areas have more separations overall (47% more), as a result of larger numbers of multiple admissions.

Of people admitted to hospital more than once in a five year period, four out of five had not moved address by the time of their second episode. Of those who moved, the most movement was within, or between, areas of similar socioeconomic status, with people from the most well off areas being the least likely to have moved.

**Conclusion:**

Postcode level and SLA level data provide a reliable, although understated, indication of socioeconomic disadvantage of area. The majority of Perth residents admitted to hospital in Western Australia had the same address when admitted again within five years. Of those who moved address, the majority had moved within, or between, areas of similar socioeconomic status.

Access to data about individuals from the Western Australian Data Linkage System shows that more people from disadvantaged areas are admitted to a hospital, and that they have more episodes of hospitalisation. Were data to be available across Australia on a similar basis, it would be possible to undertake research of greater policy-relevance than is currently possible with the existing separations-based national database.

## Background

The majority of work in Australia describing the association between the health status, use of health services and socioeconomic status of the population uses an area-based measure of socioeconomic status. It is necessary to use a proxy measure (the socioeconomic status of the population in the area) because there is no direct measure in the major administrative health record collections of the socioeconomic status of the individual about whom the event is recorded.

However, the application of an area-based measure requires a number of assumptions, including that people who move do so between, or within, geographic areas of similar socioeconomic status; and that the (often large) areas used in these analyses provide a reliable indication of the socioeconomic status and health service utilisation of the individuals in the area about whom the event is recorded. Area level socioeconomic status can also be considered as an independent predictor. For example, an individual with low socioeconomic status in an area of higher socioeconomic status is more likely to have better health outcomes than their counterpart in an area of lower socioeconomic status [1,2]. This aspect is not addressed in this paper.

In relation to this latter point, Hyndman et al [3] found that "Misclassification of individuals to SES groups based on the basis of postcode caused an underestimation of the true relationship between SES and health-related measures. A reduction of this misclassification by using smaller spatial areas, such as CD or census enumeration districts, will provide improved validity in estimating the true relationship." A reduction in strength of correlation with increasing size of area is consistent with the results of this paper. In a study of hospitalisations in Michigan, USA, Hofer et al [4] found that 'the impact of socioeconomic characteristics on hospitalization rates is consistent when measured by individual or community-level measures'. This is an encouraging finding for those limited to using area-based data.

Another limitation of the majority of Australian health-related datasets is that they record events (eg., hospital inpatient separations, services by general medical practitioners), rather than individuals.

The analysis in this report uses the Western Australian Data Linkage System to explore the reliability of area data as a proxy for socioeconomic disadvantage when analysed for the relatively large geographic units often used in health-related research: it also addresses the limitations of using data about events, rather than individuals. It does this by examining the extent to which hospital inpatient separation rates vary, both overall and by socioeconomic status of area of residence, when calculated at various levels of aggregation, from Census Collection District (CD) – the smallest area level for which a measure of socioeconomic status is available – to the larger units of postcode and Statistical Local Area (SLA). Methods applied include the calculation of correlation coefficients and examination of hospital separation rates by quintile of socioeconomic disadvantage of area, separately for events and individuals.

The report also examines the reliability of the socioeconomic status of the address over time, by examining the extent of change in socioeconomic status of area of residence for individuals with repeat hospital episodes over a five year period.

The analysis shows that aggregating data to larger area reduces the gap between the index scores for the most disadvantaged and least disadvantaged areas, with the greatest impact on the scores for the most disadvantaged areas. This results in an understatement of the extent of disadvantage in the most disadvantaged areas, as well as an understatement in inequality between the most well off and the poorest areas.

## Results

### Individuals

Over the five years from 1994 to 1998, a total of 358 948 residents of Perth were admitted to a hospital in Western Australia on one or more occasion, an average of 71 750 individuals admitted per annum. Just over half (53.6%) the individuals admitted were females; 46.4% were males.

**Figure 1:**
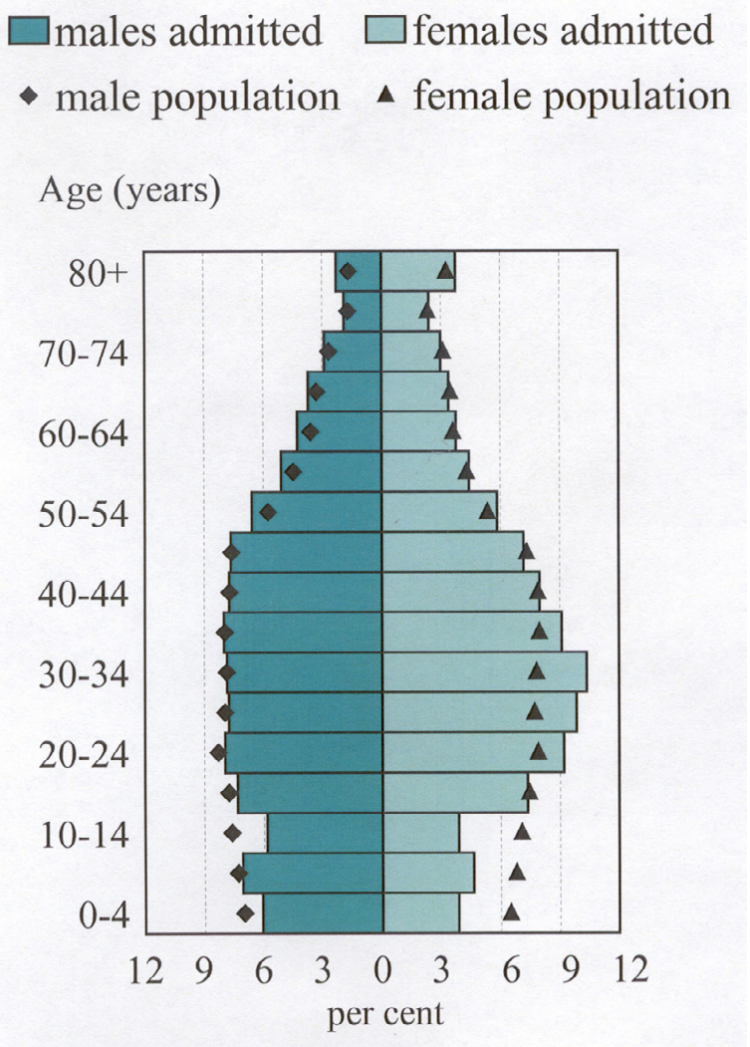
**Perth residents admitted to hospital, by age and sex at first admission, 1994–98. **Perth population is at 30 June 1996. Per cent shown is of males and females separately, not for persons.

The rate of individuals admitted was 16.4% higher for females (247.6 separations per 1000 population) than for males, (212.7 separations per 1000 population) (Table 1). As can be seen in Figure 1, the rates of males and females admitted vary notably by age. For females, the highest rate is in the 30 to 34 year age group (with a further three of the five highest female rates between ages 20 and 39 years), with the second highest rate in the 80 years and over age group. The highest male rate, in the 80 years and over age group, is substantially above the next highest male rates, in the 50 to 69 year age groups.

**Table 1:** Perth residents admitted to hospital, by age and sex, at first admission, 1994–98

*Rate per 1000*			
**Age**	**Males**	**Females**	**Persons**

0–4	185.3	147.2	166.8
5–9	207.1	168.4	188.3
10–14	163.5	135.5	149.9
15–19	201.0	244.4	222.4
20–24	204.3	288.2	245.6
25–29	207.8	315.7	261.4
30–34	212.9	328.1	270.9
35–39	214.3	282.5	248.8
40–44	213.4	250.4	232.3
45–49	214.3	242.4	228.2
50–54	243.6	270.2	256.5
55–59	242.6	254.7	248.6
60–64	252.7	257.5	255.2
65–69	240.5	241.4	241.0
70–74	232.2	237.9	235.3
75–79	237.5	254.8	247.6
80+	291.7	283.5	286.2
**Total**	**212.7**	**247.6**	**230.3**

A total of 358 768 Perth residents had one admission to a Western Australian hospital over the five years from 1994 to 1998, with a further 298 805 people admitted on two or more occasions (Table 2). The number of people with two or more admissions in any period is higher in the earlier years, as the more time that passes the greater the opportunity for a second admission. That is, those with a first admission in 1994 have had more time to record a second admission than have those with a first admission in 1995: thus the greater number with two or more admissions in 1994.

**Table 2:** Perth residents admitted to hospital, by number of admissions and year of separation, 1994–98

**Year**	**Individuals**		
	
	**One admission**	**Two or more admissions**	**Total**
1994	71 566	118 039	189 605
1995	68 400	75 830	144 230
1996	68 989	52 577	121 566
1997	71 917	34 497	106 414
1998	77 896	17 862	95 758
**Total**	**358 768**	**298 805**	**657 573**

Just over half (54.6%) those admitted to hospital had one admission over this period, and more than one third (36.0%) had between two and four admissions, together comprising 90.6% of those admitted (Table 3).

**Table 3:** Residents of Perth admitted to hospital, 1994–1998, by number of admissions per person

**Admissions per person**	**Number**	**Per cent**
1	358 769	54.6
2–4	236 611	36.0
5–9	46 377	7.1
10+	15 821	2.4
**Total**	**657 578**	**100.0**

Females accounted for just over half (53.6%) of those admitted once, compared with 59.7% of those admitted more than once. For males, the proportions were 46.4% and 40.3%, respectively.

### Separations

There were 1 665 308 separations of Perth residents from Western Australian hospitals, an average of 2.53 separations per person admitted over the five years from 1994 to 1998. Over half (55.1%) of the separations were of females and 44.9% were of males.

**Figure 2:**
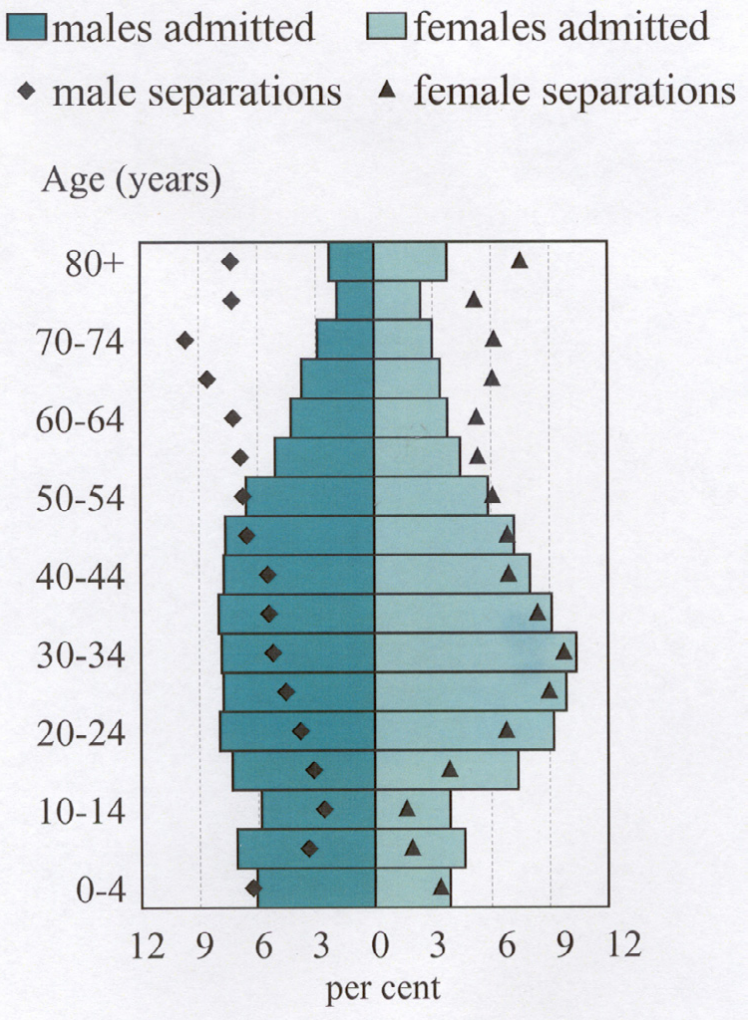
**Perth residents admitted to hospital and total separations, by age and sex, 1994–98. **Perth population is at 30 June 1996. Per cent shown is of males and females separately, not for persons.

Figure 2 shows the profiles of males and females, by age, for both individuals admitted (as in Figure 1) and separations. For males, the proportion of individuals admitted is highest at ages 20 to 49 years, dropping away at younger and older ages, with the latter exhibiting a particularly marked drop. Total separations for males are generally highest at older ages (the highest at ages 70 to 74 years), reflecting the higher number of separations per person. The notable exception is the high proportion of separations in the 0 to 4 year age group. The profile of the proportion of females admitted is similar to that for males, although it is somewhat distended at ages 20 to 39 years. The proportion of separations of females at ages 25 to 54 years closely follows that for females (individuals) admitted.

The main differences in the profiles of male and female separations are evident at the youngest ages (higher proportions of males), from ages 20 to 44 years (higher proportions of females) and from 50 to 79 years (higher proportions of males). The ages at which the highest rates of admissions of individuals and of multiple admissions (the gap between the separations and admitted profiles) occur are clearly visible in the chart.

Unlike the rates for individuals admitted (Table 1, above), the highest rates for separations of both males and females occur in the oldest age groups (Table 4). The five highest rates for both males and females are in the age groups 60 to 64 years and over, with male rates higher (and often substantially so) than female rates. Also of note is the high rate of separations for females at ages 30 to 34 years (1,672.2 admissions per 1000 population): this is the sixth highest rate for females, and is more than twice the rate for males at the same age (729.5 separations per 1000 population).

**Table 4:** Separations of Perth residents, by age and sex, 1994–98

*Rate per 1000*			
**Age**	**Males**	**Females**	**Persons**

0–4	989.4	697.9	847.6
5–9	513.2	379.4	448.0
10–14	375.3	310.5	343.8
15–19	443.7	693.4	567.2
20–24	505.2	1 151.9	823.5
25–29	630.1	1 562.6	1 093.4
30–34	729.5	1 672.2	1 203.9
35–39	747.8	1 411.6	1 083.2
40–44	780.6	1 175.8	982.2
45–49	947.6	1 253.0	1 099.0
50–54	1 289.3	1 528.1	1 405.5
55–59	1 686.8	1 666.8	1 676.9
60–64	2 210.8	1 957.6	2 082.6
65–69	2 859.5	2 356.6	2 598.8
70–74	3 991.2	2 661.3	3 268.6
75–79	4 723.2	2 979.7	3 706.7
80+	4 823.9	3 086.5	3 667.4
**Total**	**1 099.0**	**1 345.0**	**1 223.0**

## Discussion

### Effect of aggregation of areas on disadvantage scores

As noted, the majority of the analysis by socioeconomic status undertaken in the health sector in Australia is area based, and uses the postcode or SLA as the unit of analysis. This raises the question of the extent to which area based analyses at the postcode or SLA level provide a reliable indication of the socioeconomic status and health service utilisation of the individuals admitted. This report explores the reliability of postcode or SLA level data by examining the extent to which rates of individuals admitted and separations vary when calculated at various levels of aggregation (CD, postcode and SLA). Ideally, the comparison would be between the socioeconomic status of individuals and of areas; however, the smallest area level for which a measure of socioeconomic status is available is the CD.

Variation in the minimum and maximum Index of Relative Socio-economic Disadvantage (IRSD) scores when calculated at the CD, postcode and SLA level is striking and clearly shows the value of the smaller unit in area based analyses (Table 5). The range at the CD level is from a minimum index score of 532 to a maximum index score of 1221, a differential of 2.3 times. When individuals and separations are analysed by postcode, the range in the IRSD scores is narrower, from 863 to 1168 (a differential of 1.4). At the SLA level it is slightly lower again (a differential of 1.3). The effect of aggregation to the larger areas is most noticeable in the minimum IRSD score, increasing the minimum score by 70.5% from the CD level to the SLA level. At the other end of the scale, the maximum score varies little, dropping by 4.0%. That is, the greatest loss in specificity in the IRSD score is in the most disadvantaged areas.

**Table 5:** Range of IRSD scores for area of address of individuals and separations

**Variable**	**Median for individuals**	**Minimum**	**Maximum**	**Ratio: Maximum/minimum**
		**for separations**		
Collection District (1)	1012	532	1221	2.30
Postcode (2)	1015	863	1168	1.35
Statistical Local Area (3)	1017	907	1174	1.29
**Ratio of IRSD scores in area (3) to area (1)**	**1.00**	**1.70**	**0.96**	**..**

Thus, the use of larger area aggregates reduces the gap between the index scores for the most disadvantaged and least disadvantaged areas (thus lessening the extent of inequality between these areas), with the greatest impact on the scores for the most disadvantaged areas (thus understating the extent of inequality in these areas). Notably, the difference between the maximum and minimum scores, and the absolute level of the scores, is much less marked between the postcode and SLA.

There was a strong association between the IRSD scores for CDs and those for postcode of usual address at the first admission (a Spearman correlation coefficient of 0.74). The correlations were between CDs grouped to quintiles and postcodes grouped to quintiles, ranked by the IRSD, and not between individual CDs and postcodes. A weaker association was found between the quintiles for CDs and those for SLAs (0.64 for people with one separation and 0.63 for people with more than one separation) (Table 6). There was little difference in correlation coefficients for those who had moved address. Similar Spearman correlation coefficients were calculated for raw IRSD scores.

**Table 6:** Spearman correlation coefficients between IRSD of address for individuals (at first discharge) and area level

**Variable**	**Area level of first discharge**		
	
	**CD**	**Postcode**	**SLA**
Individuals:			
one separation	1.00	0.74	0.64
more than one separation	1.00	0.74	0.63
more than one separation & moved address	1.00	0.73	0.62

### Effect of aggregation of areas on separation rates

Data at the CD level for the five years from 1994 to 1998 show a variation in rates of individuals admitted from 51 442 admissions per 100 000 population in the most advantaged areas to 58 130 admissions per 100 000 population in the most disadvantaged areas (Table 7). This is a differential of 13%. The differential in separation rates is substantially higher, at 47%, reflecting multiple admissions.

**Table 7:** Residents of Perth admitted to hospital, 1994–1998, by socioeconomic disadvantage of area for selected area levels

**Quintile**	**Individuals admitted**			**Separations**		
		
	**CD**	**Postcode**	**SLA**	**CD**	**Postcode**	**SLA**
	**Number**					
Q1: Least disadvantaged	126 615	123 380	138 127	294 130	303 131	340 294
Q2	130 907	123 465	114 244	294 307	326 652	279 537
Q3	133 073	126 770	142 107	316 066	328 999	363 908
Q4	124 279	128 863	123 199	327 228	328 630	313 879
Q5: Most disadvantaged	142 704	155 100	139 901	433 577	377 896	367 690
**Total**	657 578	657 578	657 578	1 665 308	1 665 308	1 665 308
	**Rate (per 100 000 population)**					
Q1: Least disadvantaged	51 442	48 247	51 950	119 813	120 567	127 986
Q2	53 343	48 239	52 235	120 582	129 945	127 810
Q3	53 889	48 789	52 656	127 995	126 618	134 841
Q4	50 919	51 263	52 564	133 342	128 400	133 920
Q5: Most disadvantaged	58 130	61 691	58 491	176 157	147 734	153 728
**Total**	53 547	53 547	53 547	135 607	135 607	135 607
**Rate ratio: Ratio of rate in Q5 rate in Q1**	1.13***	1.28***	1.13***	1.47***	1.23***	1.20***

The extent of any inequality is shown by the rate ratio, which expresses the ratio of the rate in Quintile 5 to the rate in Quintile 1; rate ratios indicating differing significantly from 1.0 are shown with * p < 0.05; ** p < 0.01; *** p < 0.001.

When data are aggregated to postcode area or SLA, the differentials in separation rates between Quintile 5 and Quintile 1 areas are smaller (differentials of 1.23 and 1.20, respectively) than at the CD level (a differential of 1.47) (Table 7). In the case of postcodes, this is largely because of the lower separation rate in Quintile 5 areas (likely to be a result of the process of aggregating CDs), whereas for SLAs it is a combination of a lower separation rate in Quintile 5 areas and a higher rate in Quintile 1 areas (likely to be a result of the aggregation process, exacerbated by the variable size of SLAs – see section titled 'Methods, Area' under 'Methods.' The differential in rates of individuals admitted is the same for data at the SLA and CD level, but higher for postcode areas. These results again reflect the difficulty inherent in producing groups of approximately equal populations.

While just over half (54.6%) those admitted to hospital had one separation over this period, the proportion varied from 56.3% in Quintile 1 to 51.9% in Quintile 5 (Table 8). This is as expected, with people from the most disadvantaged areas representing a smaller proportion of those with one separation and a larger proportion with more than one separation.

**Table 8:** Number of separations per individual, by socioeconomic disadvantage of area, Perth residents, 1994–1998

**Separations per person**	**Quintile 1**	**Quintile 2**	**Quintile 3**	**Quintile 4**	**Quintile 5**	**Total**
	**Number**					
1	69 485	69 118	69 960	69 709	80 497	358 769
2–4	43 274	43 776	45 566	46 747	57 244	236 607
5–9	7 907	7 902	8 449	9 220	12 899	46 377
10+	2 714	2 668	2 793	3 187	4 459	15 821
**Total**	**123 380**	**123 465**	**126 770**	**128 863**	**155 100**	**657 578**
	**Per cent**					
1	56.3	56.0	55.2	54.1	51.9	54.6
2–4	35.1	35.5	35.9	36.3	36.9	36.0
5–9	6.4	6.4	6.7	7.2	8.3	7.1
10+	2.2	2.2	2.2	2.5	2.9	2.4
**Total**	**100.0**	**100.0**	**100.0**	**100.0**	**100.0**	**100.0**

There is a substantial difference in the proportion of the population in Quintiles 5 and 1 having two or more separations (a difference of 38.8%, from a rate of 30 389 separations per 100 000 persons in Quintile 5 to 21 897 separations per 100 000 persons in Quintile 1): the differential for people having one separation is lower, although still notable at 16.1% (a rate of 32 790 separations per 100 000 persons in Quintile 5 and 28 231 separations per 100 000 persons in Quintile 1) (Table 9).

**Table 9:** Separations per individual, by socioeconomic disadvantage of area, Perth residents, 1994–1998

**Separations per person**	**Quintile 1**	**Quintile 2**	**Quintile 3**	**Quintile 4**	**Quintile 5**	**Total**	**Ratio of rates in Q5/Q1**
	**Per cent**						
1	19.4	19.3	19.5	19.4	22.4	100.0	..
2+	18.0	18.2	19.0	19.8	25.0	100.0	..
**Total**	18.8	18.8	19.3	19.6	23.6	100.0	..
	**Rate per 100 000 population**						
1	28 231	28 165	28 331	28 561	32 790	29 215	1.16***
2+	21 897	22 146	23 006	24 236	30 389	24 332	1.39***
**Total**	50 128	50 311	51 337	52 797	63 180	53 547	1.26***
	**Average admissions per person with two or more admissions**						
**Number**	4.2	4.1	4.3	4.4	4.7	4.4	1.12***

The extent of any inequality is shown by the rate ratio, which expresses the ratio of the rate in Quintile 5 to the rate in Quintile 1; rate ratios differing significantly from 1.0 are shown with * p < 0.05; ** p < 0.01; *** p < 0.001.

The average number of admissions per person for people admitted to hospital on more than one occasion over the five years to 1998 was 4.4; this varied from 4.2 separations per person admitted in the least disadvantaged areas to 4.7 in the most disadvantaged areas.

### Reliability over time of address as a proxy for socioeconomic status

Studies using the address of usual residence as a proxy for socioeconomic status require two important assumptions. They are that:

• people who move do so within, or between, areas of similar socioeconomic status; and that

• the areas used in an area based analysis (which can vary in size and are quite often large) provide a reliable indication as to the socioeconomic status and use of health services of the individuals in the area.

Data from the 1996 Census show that 53.5% of Perth's population at the 1996 Census reported that they had a different address to that at the previous Census, five years earlier [5]. Data were not available to compare the IRSD of the first and last SLA of address of the Perth population who moved. However, almost one quarter (24.0%) of Perth residents who moved between the 1991 and 1996 Censuses moved to an address within the same SLA. That is, some 59.3% of the population were in the same SLA after five years (either moved within the SLA, or did not move). This is an encouraging statistic for area based analyses.

Similarly, almost four out of five people admitted to hospital more than once in a five year period had not moved (out of the CD of their address at the first separation) by the time of their second separation. For example, of the 298 809 people admitted to a Perth hospital more than once over the five year period 1994 to 1998, over three quarters (78.6%, 64 075 people) had the same address at the time of the second separation. People were recorded as having 'moved' if the CD of their address changed between the first and last separation over the period from 1994 to 1998. Movement to a different address within a CD was not included.

The following table illustrates, for people with multiple admissions, the extent of movement by socioeconomic status. For this part of the analysis, the CD of first and last separation have been allocated to quintiles of socioeconomic disadvantage of area, to provide a comparison of the extent of movement between different levels of socioeconomic status. The construction of the quintiles is described in the section titled 'Methods, Measurement of socioeconomic status' under 'Methods.'

Table 10 shows, for people who moved to an address in another CD, that:

**Table 10:** Residents of Perth admitted to hospital more than once, 1994–1998, who changed address, by socioeconomic disadvantage of area

**CD of first separation**	**CD of last separation (%)**						**Total**
	
	**Quintile 1**	**Quintile 2**	**Quintile 3**	**Quintile 4**	**Quintile 5**	**Total**	**Number**
Quintile 1	40.2	22.8	16.4	15.9	4.7	100.0	9 537
Quintile 2	21.5	24.4	22.9	23.6	7.5	100.0	10 551
Quintile 3	12.7	20.3	24.1	32.5	10.5	100.0	11 730
Quintile 4	7.8	14.6	22.0	40.3	15.3	100.0	13 298
Quintile 5	4.6	9.2	15.0	40.7	30.5	100.0	18 875
**Total**	**14.8**	**16.9**	**19.6**	**32.6**	**16.0**	**100.0**	**63,991**

• people from the most well off areas are less likely to have moved to areas of greatly different socioeconomic status (ie, changed quintiles) than are those from the most disadvantaged areas – 40.2% of people in the most advantaged areas (Quintile 1) remained there, despite moving from the CD of their first separation. The proportion in the most disadvantaged (Quintile 5) areas was a lower 30.5%;

• while there is movement right across the socioeconomic profile, most movement is between adjacent quintiles. For example, of the 18 875 people who lived in the most disadvantaged areas at their first separation (and moved before a subsequent admission), 71.2% had moved to a CD in the same or next ranked quintile (Quintiles 5 or 4), with just 4.6% moving to the most advantaged areas. Similarly, of the 9 537 people in the most well off areas at their first separation, 63.0% had moved to a CD in the same or next ranked quintile (Quintiles 1 or 2), with a similarly low proportion (4.7%) moving to the most disadvantaged areas;

• the most substantial movement between quintiles was of people moving from an address rated as Quintile 5 to one rated as Quintile 4 (40.7%); this was marginally higher than the proportions moving within Quintiles 4 or 1 (40.3% and 40.2%, respectively).

There is a strong association between the quintile of socioeconomic disadvantage of area at the first and the last discharge when analysed by CD (a correlation coefficient of 0.88) or SLA (a correlation coefficient of 0.89) of usual address (Table 11). This supports the earlier finding that people admitted to hospital who had moved between episodes, moved to or within areas of similar socioeconomic status. The weaker correlations between CD and SLA (see table) highlight the loss in specificity of the index score when aggregated to the (larger) SLA level.

**Table 11:** Correlation coefficients between quintile of socioeconomic disadvantage of area of address of first and last separation, 1994–98

**Area of address**	**CD of**		**SLA of**	
	
	**first separation**	**last separation**	**first separation**	**last separation**
**CD of first separation**	1.00	0.88	0.66	0.60
**CD of last separation**	0.88	1.00	0.60	0.65
**SLA of first separation**	0.66	0.60	1.00	0.89
**SLA of last separation**	0.60	0.65	0.89	1.00

Table includes people admitted more than once, who had moved from the CD of their address at their first separation. Area of address shown at various levels of aggregation of areas.

## Conclusions

The analysis shows that, for Perth residents admitted to hospital, the use of larger area aggregates reduces the gap between the index scores for the most disadvantaged and least disadvantaged areas, thus understating the extent of inequality between these areas. The greatest impact of aggregation of areas is on the scores for the most disadvantaged areas. This results in an understatement of the extent of disadvantage in the most disadvantaged areas, as well as an understatement in the extent of inequality between the most well off and the poorest areas.

Further, the analysis shows that a more people from the most disadvantaged areas are admitted to hospital than from the most well-off areas (13% more), and that these people have more separations overall (47% more), as a result of larger numbers of multiple admissions.

As regards the extent of movement, four out of five people admitted to hospital more than once in a five year period had not moved (out of the CD of their address at the first separation) by the time of their second separation. In addition:

• people from the most well off areas are less likely to have moved to areas of greatly different socioeconomic status than are those from the most disadvantaged areas;

• while there is movement right across the socioeconomic profile, most movement out of a quintile is to areas in adjacent quintiles; and

• the most substantial movement between quintiles was of people moving from an address rated as Quintile 5 to an address rated as Quintile 4, although this was only marginally higher than the proportions moving within Quintiles 4 or 1.

In summary, postcode level and SLA level data provide a reliable indication of socioeconomic disadvantage of area, when compared with CD-level data. That is, the association between rates of total separations and individuals admitted and socioeconomic disadvantage of area evident at the smallest area level (CD) is also evident in the higher level area aggregates of postcode and SLA.

It is reasonable to assume that similar relationships exist in other Australian cities, as well as in other health-related activity (eg. visits to general medical practitioners).

Given the widespread use in Australia of area based analyses at the postcode and SLA level, and the limitations of CDs an area level for the analysis of most health datasets, it is important to know that such analyses provide a reliable indication of the direction and underlying strength of the influence of socioeconomic factors in hospital admissions rates.

This is not to imply that the postcode or SLA are the ideal areal unit for analysis, nor that data for Collection District would be. The ideal population size for area-level analysis is likely to vary dependent on the number of cases in the dataset under analysis. For datasets with a large number of cases per capita (eg. services by general medical practitioners) the number will be smaller than those with a small number of cases per capita (eg. deaths), even with aggregation of data over a number of years. May SLAs have much larger populations than are necessary to produce reliable results; and the populations of most CDs are too small (see Table 12). HealthWIZ [6], the National Social Health Database, comprising among the most widely available small area datasets in Australia, seeks to provide health service use and health status data for areas with populations of approximately 10 000. This is a useful benchmark.

**Table 12:** Number of areas and average population for CDs, postcodes and SLAs in Perth, 1996

**Area**	**Number**	**Population**		
		
		**In smallest**	**In largest**	**Average**
CD	2,297	15	1 861	535
Postal area	105	42	49 551	11 780
SLA	37	876	103 736	33 631

It is also clear that data as to socioeconomic position at the smallest area level possible or, more importantly, of individuals, would also be of value. Were data to be available across Australia on a similar basis to that from the Western Australian Data Linkage System, it would be possible to undertake research of greater policy-relevance than is currently possible with the existing separations-based national database. Such moves are under consideration in several Australian States.

Further, linking data (eg, using probabilistic linkage) for individuals in the Western Australian Data Linkage System to the Australian Bureau of Statistics Population Census has the potential to add considerable value to such analyses. For example, it would be possible to examine an individual's characteristics of education, occupation, labour force status, housing tenure etc., and to more directly examine the relationships between the number of individuals admitted and total separations and these important socioeconomic variables. Linkage to death registration data would also be valuable in understanding more about outcomes related to socioeconomic status. This latter example is a possibility under recently announced plans for the ABS to test the linking of 2006 Census of Housing and Population data to other datasets, such as deaths registrations, held under their Act. This is similar to the approach elsewhere, including New Zealand [7]. It is to be hoped that such arrangements can be put in place in Australia in the near future.

## Methods

### Terminology

The report addresses differences in the number of individuals admitted and the number of separations they incurred. These are described as 'individuals', or individuals admitted' and separations (the total number of separations, where an individual may have had one or more episodes of hospitalisation over the period of the analysis). 'Separation' is the term describing a completed hospital episode: it is defined in the section titled 'Glossary, Separation' under 'Glossary.'

### Data sources

Details of all separations to public and private hospitals in Western Australia for the five years from 1994 to 1998 were extracted from the Western Australian Hospital Morbidity Database (HMDS). Any separation records thought to belong to the same person had previously been linked together within the Data Linkage System, permitting analyses to be performed for both separations and individual persons. The population used in calculating rates is the 1996 Census population.

The analysis has been limited to separations of residents of Perth, but includes separations occurring at any public acute or private hospitals in Western Australia.

### Area

Areas used in the analysis are the Census Collection District (CD), postcode and Statistical Local Area (SLA). See Glossary for definitions of CD, postal area and SLA.

The HMDS includes address details for each separation from a hospital in Western Australia since 1993. These addresses have been linked to a Western Australian street address database to assign northing and easting points (geo-codes). These points were then assigned to the appropriate 1991 or 1996 CD using the ABS CData96 mapping tool. The postcode and SLA of the address were then determined by allocation of CDs to postcode or SLA. The boundaries for CDs and SLAs are consistent. However, boundaries for CDs and postcodes are not, so CDs were allocated to postcodes on a 'best fit' basis (see Glossary).

Consequently, comparisons can be made between results for CDs and postcode areas, CDs and SLAs and postcode areas and SLAs. This is particularly important, as much of the area analysis undertaken in the health sector in Australia uses the postcode or the SLA, as a majority of data are only available at these area levels, and it is widely accepted that the larger the area, the less homogenous the population is likely to be.

There were 2 297 CDs in Perth at the 1996 Census, with 105 postcodes and 37 SLAs. The average population size at each of these area levels is shown in Table 12; these data emphasise the variation in size of the areas at each area level.

### Measurement of socioeconomic status

In the absence of any direct measure of socioeconomic status in the hospital inpatient data, the socioeconomic status of the area of the address of the individual admitted is used as a proxy measure. The Index of Relative Socio-Economic Disadvantage (IRSD) is the measure used to provide the socioeconomic status of the area of the address. The IRSD is one of five Socio-Economic Indexes for Areas (SEIFA) produced by the Australian Bureau of Statistics (ABS) from data collected at the 1996 Population Census. It is calculated at the CD level and can be produced for other area levels. The postcode and SLA level index scores in this report are the population weighted average of the IRSD scores for the CDs in the postcode or SLA. This calculation is undertaken for all CDs in the postcode or SLA, not just those for which hospital episodes were recorded.

Each area level (CD, postcode or SLA) was allocated to one of five groups (quintiles). For example, for SLAs, Quintile 1 comprises the SLAs with the highest IRSD scores (most advantaged areas), and Quintile 5 comprises the SLAs with the lowest IRSD score (most disadvantaged areas): each quintile comprises approximately 20% of the Perth population. This process does not provide an exact allocation of population, so the resultant populations are only 'approximately' equal, and the larger the areal units being allocated, the less likely they are to be equal. As shown in Table 13, when areas were ranked by their IRSD score at the CD level and then grouped to produce quintiles, the resultant populations were relatively close to the ideal population in each quintile of 245 607 (one fifth of 1 288 036). The quintiles based on postcode areas had rather 'lumpier' populations (greater variation around the one fifth figure of 254 859 per quintile – and a higher total of 1 274 297, due to boundary differences between CDs and postcodes. The quintiles based on SLAs were the most variable. For example, the SLA of Wanneroo – South West (with a population of 103 176) had a score marginally below the cut-off score between Quintile 1 and Quintile 2. However, the inclusion of Wanneroo – South West in Quintile 2 resulted in populations in Quintile 1 and 2 of 161 707 and 321 889, respectively. Moving Wanneroo – South West to Quintile 1 left a population of 218 713 in Quintile 2 and increased that in Quintile 1 to 265 883. While these populations are substantially different from the ideal population, they are the best that can be achieved.

**Table 13:** Population of quintiles at various area levels, 1996

**Quintile**	**CD**	**Postcode**	**SLA**
1	246 131	255 726	265 883
2	245 406	255 942	218 713
3	246 937	259 835	269 879
4	244 072	251 378	234 378
5	245 490	251 416	239 183
**Total**	**1 228 036**	**1 274 297**	**1 228 036**

### Analysis

Three (different) IRSD scores were added to each hospital separation record, based on the CD, postcode or SLA that had been previously assigned to the address on that record. It should be noted that these IRSD scores were actually the average score for the particular CD, postcode or SLA as calculated from 1996 Census data. Quintile ranks for each aggregation level were also applied using population weighting as described above.

For analyses involving multiple admissions, the IRSD value used was that for the first separation in the five-year period. These 'first' separations were isolated using the internal links between separation records for the same person and the separation date. Of course, many of these 'first' separations could have been preceded by separations occurring before 1994.

Rates are crude rates, per 100 000 population. Ideally the data would have been standardised (by the indirect method). However, access to the source data were limited and to requested tables, and standardisation was not an option.

As the data were from a complete enumeration (all admissions to hospital), confidence intervals were only calculated for measures of difference (in this case, rate ratios).

The Spearman Rank Correlation has been used in the analysis to indicate the degree of correlation between pairs of variables.

## Glossary

### CD

The Collection District (CD) is the smallest area level in the Australian Bureau of Statistics' statistical geography and is primarily an area used in the five yearly population census.

### Index of Relative Socio-Economic Disadvantage

The Index of Relative Socio-Economic Disadvantage (IRSD) is one of five Socio-Economic Indexes for Areas produced by the Australian Bureau of Statistics at recent population censuses. Produced using Principal Components Analysis, it summarises information available from variables related to education, occupation, income, family structure, race (the proportion of Indigenous people), ethnicity (poor proficiency in use of the English language) and housing. The variables are expressed as percentages of the relevant population. The IRSD is available at the Census Collection District level and was then be calculated for postcodes and SLAs by weighting the CD level scores by their population. The IRSD is calculated to show the relativity of areas to the Australian average for the particular set of variables which comprise it; this average score is set at 1000. Scores below 1000 indicate areas with relative disadvantaged populations under this measure, and scores above 1000 indicate areas with relatively advantaged populations. The IRSD scores at the Census Collection District (CD) level have been grouped to postal area, an area developed by ABS for the presentation of population counts and other Census data from the five-yearly population censuses to approximate postcode areas, as the ABS does not collect the postcode at the Census.

### Separation

The term describing a completed hospital episode is a 'separation'. At the time of admission to hospital, the age, sex, address of usual residence and other personal details of the patient are recorded. At the end of the episode, at the time of separation from hospital, details of the episode itself are recorded, including the date, time and method of separation (discharge, death or transfer of a patient to another care setting eg. hospital, nursing home). Consequently, hospital inpatient data collections are based on separations.

### Postal area

The postal area is an area developed by ABS for the presentation of population counts and other Census data from the five-yearly population censuses. It approximates postcode areas, as the ABS does not collect the Australia Post postcode at the Census. Postal areas comprise Census Collection Districts (CDs) grouped to approximate postcode areas. Where a CD does not fit entirely within a postcode area, it is allocated to the postcode area into which the population largely falls. Where a CD covers more than one postcode area, the total CD population is allocated to one postcode.

The IRSD scores at the Census Collection District (CD) level have been allocated to postal areas as described in the section titled 'Methods, Index of Relative Socio-Economic Disadvantage' under 'Methods.'. Similarly, the postal area of each separation was approximated from the CD of the address.

The term postcode, rather than postal area, is used in the text, for ease of reading.

### Postcode

See postal area, above.

### Quintile of socioeconomic disadvantage of area

See section titled 'Methods, Measurement of socioeconomic status' under 'Methods.'

### SLA

An SLA in Perth is generally equivalent to a local government area, with additional codes allocated to local government areas split for statistical purposes (mainly local government areas with large populations, split to form SLAs with smaller populations).
